# Rate of perfusion modulates colloidal carbon leakage from rat intestinal microvessels *in vitro*

**DOI:** 10.1155/S096293519500055X

**Published:** 1995-09

**Authors:** A. M. Northover, B. J. Northover

**Affiliations:** Department of Pharmaceutical Sciences School of Applied Sciences De Montfort University Leicester LE1 9BH UK

## Abstract

In order to investigate the effects of varying the rate of flow on endothelial integrity the rat isolated small intestinal vasculature was perfused at 1, 5, 10 or 20 ml/min with a gelatin-containing physiological salt solution (GPSS), followed by an injection of colloidal carbon suspension (CC). Significantly greater microvascular CC leakage occurred at 1 or 5 ml/min than at 10 or 20 ml/ mitt. CC leakage at the two slower rates of flow was reduced by adding red blood cells to the GPSS, suggesting that the microvascular endothelium became hypoxic when perfused with GPSS at 1 or 5 ml/min. After perfusion at 20 ml/min with GPSS containing resiniferatoxin (1 μM) or 5-hydroxytryptamine (100 μM), CC leakage was significantly lower than after similar perfusion at 10 ml/min. Two nitric oxide (NO) synthesis blockers, N-nitro-L-arginine methyl ester (L-NAME, 100 μM) and methylene blue (20 μM), and an NO scavenger CPTIO (100 μM) each increased CC leakage. This suggests that NO was being produced at perfusion rates of 10 or 20 ml/min. Sodium nitroprusside (10 μM), 8-bromo-cGMP (100 μM) and BN52021 (10 μM) each significantly reduced CC leakage in the presence of L-NAME.


Mediators of Inflammation 4, 344–349 (1995)

### Rate of perfusion modulates colloidal carbon leakage from rat intestinal microvessels *in vitro*

A. M. Northover[CA] and B. J. Northover

Department of Pharmaceutical Sciences, School of Applied Sciences, De Montfort University, Leicester, LE1 9BH, UK

[CA]Corresponding Author

**In order to investigate the effects of varying the rate of flow on endothelial integrity the rat isolated small intestinal vasculature was perfused at 1, 5, 10 or 20 ml/min with a gelatin-containing physiological salt solution (GPSS), followed by an injection of colloidal carbon suspension (CC). Significantly greater microvascular CC leakage occurred at 1 or 5 ml/min than at 10 or 20 ml/min. CC leakage at the two slower rates of flow was reduced by adding red blood cells to the GPSS, suggesting that the microvascular endothelium became hypoxic when perfused with GPSS at 1 or 5 ml/min. After perfusion at 20 ml/min with GPSS containing resiniferatoxin (1 μM) or 5-hydroxytryptamine (100 μM), CC leakage was significantly lower than after similar perfusion at 10 ml/min. Two nitric oxide (NO) synthesis blockers, *N*-nitro-L-arginine methyl ester (L-NAME, 100 μM) and methylene blue (20 μM), and an NO scavenger CPTIO (100 μM) each increased CC leakage. This suggests that NO was being produced at perfusion rates of 10 or 20 ml/min. Sodium nitroprusside (10 μM), 8-bromo-cGMP (100 μM) and BN52021 (10 μM) each significantly reduced CC leakage in the presence of L-NAME.**

**Key words:** BN52021, Colloidal carbon, Hypoxia, Microvascular permeability, *N*-nitro-L-arginine methyl ester, Nitric oxide, Perfusion rate, Platelet-activating factor, Red blood cells.

### Introduction

Colloidal carbon (CC) has been used routinely in this laboratory as a marker for assessing acute inflammatory responses to various compounds in the microvasculature of rat small intestinal villi during perfusion *in vitro*.[1–5] However, small amounts of CC leak even in control preparations. Tissue hypoxia, due to the relatively low oxygen-carrying capacity of the artificial perfusate being used, was one possible explanation.[1] We now report the effect on CC leakage of varying the rate of perfusion and of increasing the oxygen-carrying capacity of the perfusate by addition of pig washed red blood cells (RBCs). The possibility that the endothelium might be producing an endogenous anti-inflammatory substance such as nitric oxide, particularly at higher rates of perfusion, has been investigated, along with possible associated alterations in the release of platelet-activating factor (PAF).

### Materials and Methods

*Perfusion of rat isolated mesentery and small intestine:* The method used has been described in detail previously.[1] Rats were killed by inhalation of chloroform vapour and the anterior mesenteric artery cannulated. The mesentery with the small intestine still attached was removed to a bath of gelatin-containing physiological salt solution (GPSS) with RBCs added where appropriate (GPSS + RBC) at 37°C. Rat blood was flushed from the vasculature using 50 ml of either GPSS or GPSS + RBC delivered at a rate of 10 ml/min. Re-circulation of the perfusate was then commenced at flow rates of 1, 5, 10 or 20 ml/min for 15 min, with perfusion pressures during the last 5 min being recorded. Then 0.5 ml of CC suspension was injected near to the cannula and the perfusate thereafter allowed to run to waste. The volume of perfusate used for flushing CC from the vessel lumen was 80 ml in all cases. Flushing began at the rate of flow under specific investigation, but the last 40 ml of perfusate was always delivered at 20 ml/min to ensure complete clearance of CC from the lumen. This represents only a slight departure from the protocol adopted in previous work.[1–5] Where GPSS was used as a perfusate, 2 ml of rat washed RBCs were injected into the cannula after flushing out CC to provide a visual check on vascular patency during subsequent microscopic examination. The prepara-

© 1995 Rapid Science Publishers tion was removed from the organ bath at this point and placed in a bath of cold normal saline for further dissection of the intestine.

Where appropriate, 5-hydroxytryptamine (5HT, 100 μM) or resiniferatoxin (RFX, 1 μM), was added to the perfusate for the last 5 min of the 15 min period of re-circulation. *N*-Nitro-L-arginine methyl ester HCl (L-NAME, 20 μM, 100 μM, or 500 μM), *N*-nitro-D-arginine methyl ester HCl (D-NAME, 100 μM), 2-(4-carboxyphenyl)-4,4,5,5-tetramethylimidazoline-1-oxyl-3-oxide (CPTIO, 100 μM) or methylene blue (20 μM) were included in the perfusate from the start of the 15 min period of re-circulation. However, those compounds being tested as possible antagonists to the effects of L-NAME, i.e. 8-bromo-cGMP (8BrcGMP, 500 μM), sodium nitroprusside (SNP, 10 μM) or BN52021 (10 μM), were added to the perfusate starting 2 min prior to adding the L-NAME.

*Preparation of the intestine for image analysis:* Segments of small intestine were opened lengthways and stapled to xylene-resistant plastic coverslips. After fixation, dehydration and clearance in xylene, villi microvessels were photographed at ×100 magnification. The resulting negative micrographs were subjected to image analysis. The area depicting CC deposits was expressed as a percentage of the total area of each micrograph, as described earlier.[1–5]

*Statistical analysis:* Bonferroni's test was used for comparing multiple groups with a single control group.

*Perfusates:* GPSS had the following composition (mM): NaCl, 138; KCl, 5; $NaHCO_3$, 10.1; $MgCl_2$, 1.06; $NaH_2PO_4$, 0.416; $CaCl_2$, 2; glucose, 10; plus 2% gelatin, giving pH 7.4. Pig washed RBCs (20% v/v for flow rates of 1 or 5 ml/min, or 10% v/v for flow rates of 10 or 20 ml/min) were added as required (GPSS + RBC). Perfusates were gassed throughout with 95% $O_2$ and 5% $CO_2$.

*Chemicals used:* 5HT, RFX, L-NAME, D-NAME and 8BrcGMP were all obtained from Sigma Chemical Co., Poole, UK; methylene blue was obtained from Hopkin & Williams, Chadwell Heath, UK; SNP was obtained from David Bull Laboratories, Warwick, UK; CPTIO was obtained from Tocris Cookson, Bristol, UK; BN52021 was kindly given by Dr P. Braquet, Henri Beaufors Research Laboratories, Le Plessis Robinson, France. RFX and BN52021 were initially dissolved in dimethylsulphoxide. The latter solvent was biologically inert at the final concentration used in the perfusate. All other compounds were prepared as aqueous solutions. The dose volume added to the reservoir of perfusate was 0.2 ml in each case. All solutions containing SNP were protected from light.

Gelatin was obtained from Thornton Ross, Huddersfield, UK; Thermanox[R] xylene-resistant coverslips were obtained from Nunc Inc., Naperville, IL, USA; colloidal carbon suspension (Gunther Wagner, batch C11/1431a) was obtained from Pelikan Inks, Hanover, Germany.

### Results

*Effects of altering the rate of perfusion:* When the intestinal vasculature was perfused with GPSS at either 1 or 5 ml/min there was a significant leakage of CC into the microvessel walls in the villi (Fig. 1). At higher rates of flow (10 or 20 ml/min) the leakage of CC was significantly less (Fig. 1). Perfusion pressures rose progressively with increasing rates of flow (Table 1), but they remained more or less constant over the 5 min period of measurement at any given rate of perfusion (Table 1).

Significant further leakage of CC occurred when either 5HT (100 μM) or RFX (1 μM) was added to the GPSS perfusate at 10 ml/min during the 5 min immediately prior to injection of CC (Table 2), confirming earlier reports.[2–4] When perfusion was conducted at 20 ml/min, however, the leakage of CC in the presence of these two compounds was not significantly different from control values at that rate of flow (Table 2). Perfusion pressures were increased significantly in the presence of 5HT at both rates of flow, but not in the presence of RFX (Table 1).

*Effects of adding RBCs to the perfusate:* The above results suggested that tissue hypoxia, due to a rather low oxygen-carrying capacity of GPSS, particularly when used at perfusion rates of only 1 or 5 ml/min, might be responsible for CC leakage seen under these control conditions. When RBCs were added to GPSS (Fig. 1) the leakage of CC at flow rates of 1 or 5 ml/min was reduced to the levels akin to those recorded in the absence of RBCs at two higher rates of flow (10 or 20 ml/min). This confirmed that indeed hypoxia was a factor in promoting CC leakage at the two lower perfusion rates. At 10 or 20 ml/min, however, addition of RBCs to GPSS had no significant effect on the leakage of CC, suggesting that tissue hypoxia did not occur even in the absence of RBCs. As expected, perfusion pressures (PP 10 values) recorded in the presence of RBCs were higher than with GPSS alone and are given in Table 1. This reflects the viscosity-enhancing effect of the RBCs.

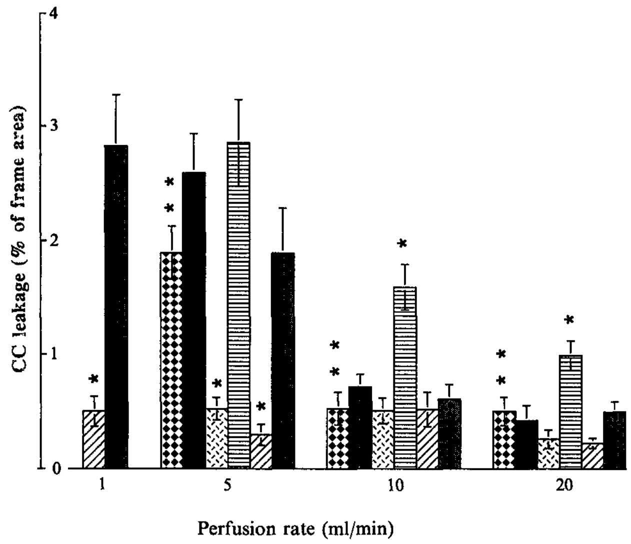

FIG. 1. CC leakage assessed by image analysis and expressed as % of frame area (see Materials and Methods). *$p < 0.05$ (Bonferroni's test) compared with GPSS alone at the same perfusion rate. **$p < 0.05$ (Bonferroni's test) compared with GPSS + L-NAME at the same perfusion rate. Results are given as mean ± S.E.M. $n = 5$ to 12. ▦, GPSS + BN (10 μM) + L-NAME (100 μM); ■, GPSS + BN (10 μM); ▨, GPSS + RBC + L-NAME (100 μM); ▤, GPSS + L-NAME (100 μM); ▨, GPSS + RBC; ■, GPSS.

*Possible involvement of NO:* Figure 1 shows that L-NAME (100 μM), an antagonist of NO production,[6] increased CC leakage in microvessels perfused with GPSS at 5, 10 or 20 ml/min. At 20 ml/min, in the presence of either a five-fold lower (20 μM) or higher (500 μM) concentration of L-NAME, CC leakage was not significantly different from control values (Table 3). This suggests that 20 μM was too low a concentration to inhibit NO synthase. Possibly L-NAME at 500 μM evoked non-specific vascular leakage effects, in addition to blocking NO synthesis. D-NAME (100 μM), which is known to be inactive as an inhibitor of NO synthesis,[6] did not alter CC leakage (Table 2).

NO is thought to bring about vasorelaxation by activating guanylyl cyclase.[7] Leakage of CC was reduced when either SNP, an activator of guanylyl cyclase,[8] or 8BrcGMP, a membrane-permeant activator of cGMP-dependent kinase,[9] was added to the GPSS just prior to adding L-NAME (Table 3). These results are consistent with a significant production of NO occurring in the absence of L-NAME. Methylene blue, which is an NO synthase inhibitor[10] as well as being an antagonist of guanylyl cyclase,[11] also increased CC leakage (Table 2). This again is consistent with a release of NO

Table 1. Effects of changing flow rates on perfusion pressures (PP) recorded after 10 min perfusion with GPSS or GPSS + RBC (PP 10), and effects of adding 5HT (100 μM) or RFX (1 μM) to the perfusate for the subsequent 5 min (PP change)[a]

| Flow rate | | *n* | GPSS | *n* | GPSS + RBC[b] | *n* | GPSS + 5HT | *n* | GPSS + RFX |
|---|---|---|---|---|---|---|---|---|---|
| 1 ml/min | PP 10 | 5 | 10.60 ± 2.62 | 4 | 28.75 ± 5.12 | | | | |
| | PP change | 5 | 0.40 ± 0.51 | 4 | 2.75 ± 1.60 | | | | |
| 5 ml/min | PP 10 | 6 | 22.67 ± 1.45 | 4 | 101.50 ± 24.46 | | | | |
| | PP change | 6 | −0.33 ± 0.21 | 4 | 5.50 ± 2.99[c] | | | | |
| 10 ml/min | PP 10 | 7 | 37.14 ± 1.96 | 5 | 68.40 ± 10.17 | 5 | 40.40 ± 2.84 | 4 | 47.25 ± 8.05 |
| | PP change | 7 | −0.71 ± 0.64 | 5 | 2.85 ± 1.31 | 5 | 38.60 ± 8.96[d] | 4 | 3.00 ± 3.94 |
| 20 ml/min | PP 10 | 12 | 85.17 ± 6.10 | 5 | 132.60 ± 21.85 | 5 | 73.00 ± 2.88 | 4 | 78.50 ± 6.31 |
| | PP change | 12 | 6.83 ± 2.49 | 5 | 7.40 ± 7.17 | 5 | 90.20 ± 8.38[d] | 4 | 16.75 ± 9.51 |

[a]Results (mm Hg) are given as mean ± S.E.M. [b]20% v/v RBC added at 1 and 5 ml/min, but 10% v/v RBC at 10 and 20 ml/min. [c]$p < 0.05$, [d]$p < 0.005$ (Bonferroni's test) compared with PP change using GPSS alone at the same flow rate.

Table 2. Effects on CC leakage in rat small intestinal villi microvessels of adding various agents to the GPSS perfusing the vasculature at 10 or 20 ml/min

| Treatment | Amount of CC leakage (as % of frame area[a])[b] | | | |
|---|---|---|---|---|
| | *n* | 10 ml/min | *n* | 20 ml/min |
| GPSS (control) | 7 | 0.61 ± 0.13 | 12 | 0.50 ± 0.09 |
| GPSS + 5HT (100 μM) | 5 | 1.88 ± 0.37[c] | 5 | 0.42 ± 0.08 |
| GPSS + RFX (1 μM) | 4 | 1.97 ± 0.44[c] | 5 | 0.57 ± 0.14 |
| GPSS + MB (20 μM) | | - | 6 | 1.38 ± 0.24[c] |
| GPSS + D-NAME (100 μM) | | - | 6 | 0.48 ± 0.08 |
| GPSS + CPTIO (100 μM) | 6 | 1.58 ± 0.26[c] | | - |

Abbreviation not in text: MB, methylene blue. [a]Each frame represents one negative micrograph magnified approximately 20 times. [b]Values are given as mean ± S.E.M. [c]$p < 0.05$, Bonferroni's test compared with the GPSS (control) value at the same flow rate.

Table 3. Effects on CC leakage in rat small intestinal villi microvessels of adding L-NAME to the GPSS perfusing the vasculature at perfusion rates of 10 or 20 ml/min, and their modification by various agents

| Treatment | L-NAME concentration (μM) | Amount of CC leakage (as % of frame area[a])[b] | | | |
|---|---|---|---|---|---|
| | | *n* | 10 ml/min | *n* | 20 ml/min |
| GPSS (Control) | – | 7 | 0.61 ± 0.13 | 12 | 0.50 ± 0.09 |
| GPSS | 20 | | – | 7 | 0.73 ± 0.19 |
| GPSS | 100 | 4 | 1.59 ± 0.20[c] | 12 | 0.99 ± 0.13[c] |
| GPSS | 500 | | – | 6 | 0.70 ± 0.21 |
| GPSS + SNP (10 μM) | 100 | 6 | 0.58 ± 0.11[d] | | – |
| GPSS + 8BrcGMP (100 μM) | 100 | | – | 5 | 0.50 ± 0.07[d] |

[a]Each frame represents one negative micrograph magnified approximately 20 times. [b]Results are given as mean ± S.E.M. [c]$p < 0.05$ (Bonferroni's test) compared with the GPSS (control) value at the same flow rate. [d]$p < 0.05$ (Bonferroni's test) compared with GPSS + L-NAME (100 μM) value at the same flow rate.

occurring during perfusion in the absence of L-NAME. The addition of CPTIO, an NO scavenger,[12] also promoted CC leakage, thus providing further evidence for the release of NO (Table 2).

*Possible involvement of PAF:* Addition to GPSS perfusate of BN52021 (10 μM), an antagonist of the effects of PAF,[13] prior to starting exposure to L-NAME, significantly reduced the subsequent leakage of CC at 5, 10 and 20 ml/min. (Fig. 1). In the absence of L-NAME, BN52021 had no effect on CC leakage. Thus, PAF appeared not to be involved when NO synthase was still operational, but may have been involved in the absence of NO.

### Discussion

The reduced CC leakage found in response to the addition of either RFX or 5HT at a perfusion rate of 20 ml/min, compared with that seen at 10 ml/min, suggested that at the higher rate of flow an endothelial cell (EC) protectant substance was being released in greater than normal amounts, or that at the lower rate of flow a leakage-promoting substance was being released. Several workers have suggested that endothelium-derived relaxant factor (EDRF), which is probably NO,[7] is released in greater than normal amounts at high rates of vascular flow[14,15] or wall shear stress.[16,17] Administration of L-NAME in our experiments was an attempt to inhibit endogenous NO production. However, the resultant increase in CC leakage was similar at 5, 10 and 20 ml/min, suggesting that even at 5 ml/min NO secretion was occurring. Hence the difference between responses seen in the present experiments at 10 and 20 ml/min more likely was due to reduced production of a leakage-promoting substance at the higher rate of flow than to some variation in the rate of NO production.

Some previous workers have shown a vascular protective role for NO,[18–21] although a pro-inflammatory role[22,23] and a dual action also have been reported.[24,25] Since these results were obtained from different vascular beds in different species the physiological role of NO remains ambiguous and its likely effects are, therefore, difficult to predict. Usually NO is considered to relax vascular tissues by activating guanylyl cyclase.[7] Three of our findings are relevant in this connection. Firstly, 8BrcGMP, given prior to L-NAME, reduced subsequent CC leakage. Secondly, treatment with methylene blue caused additional CC leakage. Thirdly, the NO scavenger CPTIO also increased CC leakage. Each of these observations is consistent with NO being involved as a normal preventer of background leakage in this vascular bed, at least during GPSS perfusion. However, $PGI_2$ also is known to be released by ECs under conditions of high shear stress,[26–28] and ATP secretion is known to be stimulated by increases in perfusion rate.[29] Since both $PGI_2$ and ATP are vasorelaxant substances, they also may be acting as endogenous CC leakage inhibitors.

PAF is known to be generated by ECs in the presence but not in the absence of L-NAME.[21] In the present experiments pre-treatment with the PAF inhibitor BN52021 inhibited CC leakage induced by L-NAME, but in the absence of L-NAME the same concentration of BN52021 failed to reduce CC leakage (Fig. 1). Presumably the production of PAF by vascular ECs can be suppressed by the NO that is normally secreted. Alternatively the EC-contracting effect, and hence the leakage-promoting effect, of normal amounts of PAF may be counterbalanced by a relaxant effect from a concomitant secretion of NO.

At the two lower perfusion rates used here, CC leakage in the absence of RBCs probably was due to tissue hypoxia. Large arteries that are exposed to hypoxia have been reported previously to release an endothelium-dependent vasoconstrictor substance,[30] and to develop inherent muscular tone.[31] Since the increased permeability to CC that was seen in the present experiments probably was brought about by retraction of the margins of previously adjacent microvascular ECs, resulting in the formation of intercellular gaps,[32] the release of an EC-contractile agent might explain the increase in CC leakage during hypoxia. In the absence of L-NAME, however, pre-treatment with BN52021 did not reduce CC leakage, so it seems unlikely that PAF was being formed during hypoxia as long as NO was available. ECs are known to release endothelins, particularly during hypoxia,[33,34] and therefore are further possible mediators of the CC leakage seen under these circumstances. Alternatively, hypoxia may cause EC retraction by increasing endothelial intracellular $Ca^{2+}$ levels directly.[35] Further work will be required to substantiate or refute these suggestions.

In the presence of RBCs there was very little CC leakage in response to L-NAME, suggesting that NO may be unimportant physiologically. It is unlikely, however, that all vascular effects of NO are precluded by normoxia since L-NAME caused significant CC leakage with GPSS as the perfusate, even at 20 ml/min. More probably, therefore, in the presence of RBCs, any NO is scavenged by haemoglobin,[36,37] perhaps along with other free radicals.[36] It is necessary to recognise, therefore, that using an RBC-free perfusate may yield experimental results that are different from those that would be derived from a normally blood-perfused vascular bed.

In conclusion, the present work suggests that at both high and low rates of perfusion with GPSS there is a release of NO, with significant effects on the permeability of the endothelial lining of villi microvessels. It is possible that at low rates of perfusion with GPSS a raised intracellular $Ca^{2+}$ concentration in the ECs occurs as a result of hypoxia, and that this stimulates NO production. At higher rates of perfusion with GPSS, however, increased shear stresses on the endothelium may serve the same function. Secretion of PAF appears to be involved in CC leakage only where NO production is inhibited. Microvascular leakage of CC is increased under conditions of limited oxygen supply, and this seems to occur when GPSS is flowing at rates of $<10$ ml/min. Increasing the oxygen-carrying capacity of GPSS by adding RBCs significantly decreased the leakage of CC. At perfusion rates higher than this, however, even in the absence of RBCs, CC leakage was low. This suggests that an adequate oxygen delivery with GPSS was achieved at higher flow rates. At each perfusion rate investigated a complex mixture of vasoactive agents appears to be involved. Further investigations are required to elucidate the nature of this mixture.

### References

1. Northover AM. An *in vitro* method for assessing the effects of pro-inflammatory and anti-inflammatory compounds on microvascular permeability in the rat small intestine. *J Pharmacol Toxicol Meth* 1993; **29:** 227–232.
2. Northover AM, Northover BJ. Involvement of protein kinase C in the control of microvascular permeability to colloidal carbon. *Agents Actions* 1993; **39:** 132–136.
3. Northover AM, Northover BJ. Lectin-induced increase in microvascular permeability to colloidal carbon *in vitro* may involve protein kinase C activation. *Agents Actions* 1994; **41:** 136–139.
4. Northover AM, Northover BJ. Stimulation of protein kinase C activity may increase microvascular permeability to colloidal carbon via α-isoenzyme. *Inflammation* 1994; **18:** 481–487.
5. Northover AM, Northover BJ. Possible bi-directional link between $ET_A$ receptors and protein kinase C in rat blood vessels. *Mediators of Inflammation* 1995; **4:** 55–59.
6. Rees DD, Palmer RMJ, Schulz R, Hodson HF, Moncada S. Characterization of three inhibitors of endothelial nitric oxide synthase *in vitro* and *in vivo*. *Br J Pharmacol* 1990; **101:** 746–752.
7. Moncada S, Palmer RMJ, Higgs EA. Nitric oxide: physiology, pathophysiology, and pharmacology. *Pharmacol Rev* 1991; **43:** 109–142.
8. Rapoport RM, Murad F. Endothelium-dependent and nitrovasodilator-induced relaxation of vascular smooth muscle: role of cyclic GMP. *J Cyclic Nucleotide Res* 1983; **9:** 281–296.
9. Lincoln TM. Effects of nitroprusside and 8-bromo-cyclic GMP on the contractile activity of the rat aorta. *J Pharmacol Exp Ther* 1983; **224:** 100–107.
10. Mayer B, Brunner F, Schmidt K. Inhibition of nitric oxide synthesis by methylene blue. *Biochem Pharmacol* 1993; **45:** 367–374.
11. Gruetter CA, Barry BK, McNamara DB, Gruetter DY, Kadowitz PJ, Ignarro LJ. Relaxation of bovine coronary artery and activation of coronary arterial guanylate cyclase by nitric oxide, nitroprusside and a carcinogenic nitrosoamine. *J Cyclic Nucleotide Res* 1979; **5:** 211–224.
12. Akaike T, Yoshida M, Miyamoto Y, *et al.* Antagonistic action of imidazolineoxyl *N*-oxides against endothelium-derived relaxing factor/•NO through a radical reaction. *Biochemistry* 1993; **32:** 827–832.
13. Braquet P, Godfroid JJ. PAF-acether specific binding sites. 2. Design of specific antagonists. *Trends Pharmacol Sci* 1986; **7:** 397–403.
14. Pohl U, Holtz J, Busse R, Bassenge E. Crucial role of endothelium in the vasodilator response to increased flow *in vivo*. *Hypertension* 1986; **8:** 37–44.
15. Rubanyi GM, Romero JC, Vanhoutte PM. Flow-induced release of endothelium-derived relaxing factor. *Am J Physiol* 1986; **250:** H1145–H1149.
16. Korenaga R, Ando J, Tsuboi H, *et al.* Laminar flow stimulates ATP- and shear stress-dependant nitric oxide production in cultured bovine endothelial cells. *Biochem Biophys Res Comm* 1994; **198:** 213–219.
17. Kuchan MJ, Jo H, Frangos JA. Role of G proteins in shear stress-mediated nitric oxide production by endothelial cells. *Am J Physiol* 1994; **267:** C753–C758.
18. Kubes P, Granger DN. Nitric oxide modulates microvascular permeability. *Am J Physiol* 1992; **262:** H611–H615.
19. Filep JG, Földes-Filep E. Modulation by nitric oxide of platelet-activating factor-induced albumin extravasation in the conscious rat. *Br J Pharmacol* 1993; **110:** 1347–1352.
20. Kurose I, Fukumura D, Miura S, *et al.* Nitric oxide mediates vasoactive effects of endothelin-3 on rat mesenteric microvascular beds *in vivo*. *Angiology* 1993; **44:** 483–490.
21. László F, Whittle BJR, Moncada S. Interactions of constitutive nitric oxide with PAF and thromboxane on rat intestinal vascular integrity in acute endotoxaemia. *Br J Pharmacol* 1994; **113:** 1131–1136.
22. Mayhan WG. Nitric oxide accounts for histamine-induced increases in macromolecular extravasation. *Am J Physiol* 1994; **266:** H2369–H2373.
23. Fujii E, Irie K, Uchida Y, Tsukahara F, Muraki T. Possible role of nitric oxide in 5-hydroxytryptamine-induced increase in vascular permeability in mouse skin. *Naunyn-Schmiedeberg's Arch Pharmacol* 1994; **350:** 361–364.
24. Laszlo F, Whittle BJR, Moncada S. Time-dependent enhancement or inhibition of endotoxin-induced vascular injury in rat intestine by nitric oxide synthase inhibitors. *Br J Pharmacol* 1994; **111:** 1309–1315.
25. Miller MJS, Clark DA. Nitric oxide synthase inhibition can initiate or prevent gut inflammation: role of enzyme source. *Agents Actions* 1994; **41**(special issue): C231–C232.
26. Grabowski EF, Jaffe EA, Weksler BB. Prostacyclin production by cultured endothelial cell monolayers exposed to step increases in shear stress. *J Lab Clin Med* 1985; **105:** 36–43.
27. Frangos JA, Eskin SG, McIntire LV, Ives CL. Flow effects on prostacyclin production by cultured human endothelial cells. *Science* 1985; **227:** 1477–1479.
28. Carosi JA, Eskin SG, McIntire LV. Cyclical strain effects on production of vasoactive materials in cultured endothelial cells. *J Cell Physiol* 1992; **151:** 29–36.
29. Hassêssian H, Bodin P, Burnstock G. Blockade by glibenclamide of the flow-evoked endothelial release of ATP that contributes to vasodilatation in the pulmonary vascular bed of the rat. *Br J Pharmacol* 1993; **109:** 466–472.
30. Rubanyi GM, Vanhoutte PM. Hypoxia releases a vasoconstrictor substance from the canine vascular endothelium. *J Physiol* 1985; **364:** 45–56.
31. Wanstall JC, Hughes IE, O'Donnell SR. Evidence that nitric oxide from the endothelium attenuates inherent tone in isolated pulmonary arteries from rats with hypoxic pulmonary hypertension. *Br J Pharmacol* 1995; **114:** 109–114.
32. Majno G, Shea SM, Leventhal M. Endothelial contraction induced by histamine-type mediators. An electron microscope study. *J Cell Biol* 1969; **42:** 647–672.
33. Gertler JP, Ocasio VH. Endothelin production by hypoxic human endothelium. *J Vasc Surg* 1993; **18:** 178–184.
34. Kourembanas S, McQuillan LP, Leung GK, Faller DV. Nitric oxide regulates the expression of vasoconstrictors and growth factors by vascular endothelium under both normoxia and hypoxia. *J Clin Invest* 1993; **92:** 99–104.
35. Arnould T, Michiels C, Alexandre I, Remacle J. Effect of hypoxia upon intracellular calcium concentration of human endothelial cells. *J Cell Physiol* 1992; **152:** 215–221.
36. Mittal CK, Arnold WP, Murad F. Characterization of protein inhibitors of guanylate cyclase activation from rat heart and bovine lung. *J Biol Chem* 1978; **253:** 1266–1271.
37. Martin W, Villani GM, Jothianandan D, Furchgott RF. Selective blockade of endothelium-dependent and glyceryl trinitrate-induced relaxation by hemoglobin and by methylene blue in the rabbit aorta. *J Pharmacol Exp Ther* 1985; **232:** 708–716.

**Received 4 May 1995;**
**accepted in revised form 13 June 1995**
